# Recurrent lateral patella dislocation affects knee function as much as ACL deficiency – however patients wait five times longer for treatment

**DOI:** 10.1186/s12891-019-2689-7

**Published:** 2019-07-08

**Authors:** Truls Martin Straume-Næsheim, Per-Henrik Randsborg, Jan Rune Mikaelsen, Einar Andreas Sivertsen, Brian Devitt, Lars-Petter Granan, Asbjørn Årøen

**Affiliations:** 1 The Department of Orthopaedic Surgery, Akershus University Hospital, 1478 Lørenskog, Norway; 2 Oslo Sports Trauma Research Center, Department of Sports Medicine, Norwegian School of Sport Sciences, Oslo, Norway; 3 Department of Orthopaedic Surgery, Lovisenberg Diaconal Hospital, Oslo, Norway; 4 OrthoSport Victoria, Melbourne, Australia; 5 Oslo University Hospital, Oslo, Norway; 6 Institute of Clinical Medicine University of Oslo, Campus Ahus. Clinic of Orthopaedic Surgery, Akershus University Hospital, Nordbyhagen, Norway

**Keywords:** Patella dislocation, Young adults and adolescents, Symptoms and function evaluation, Indication for surgery, Time to surgery, Comparative study

## Abstract

**Background:**

Surgical treatment of young patients with recurrent lateral patella dislocation (RLDP) is often recommended because of loss of knee function that compromises their level of activity or even their daily life functioning. This situation is comparable to young patients with an anterior cruciate ligament (ACL) rupture. The purpose of this study was therefore to explore the time from injury to surgery and the pre-operative symptoms and knee function of young RLPD patients scheduled for stabilizing surgery and compare this group to age and sex-matched ACL-deficient patients.

**Method:**

Forty-seven patients with unilateral RLPD listed for isolated medial patellofemoral ligament reconstruction were included in the study (RLPD-group). This group was compared to an age, sex and BMI matched ACL patient group obtained from the Norwegian knee ligament registry (ACL-group) for the following outcome measures: the knee injury and osteoarthritis outcome score (KOOS) assessed on the day of surgery and time from injury to surgery.

**Results:**

The RLPD-group scored significantly lower than the ACL-group for the three KOOS subscales “Pain” (73.6 vs. 79.8, *p* < 0.05), “Symptoms” (71.7 vs. 79.3, p < 0.05) and “ADL” (84.7 vs 89.5, p < 0.05). The lowest KOOS values were found for Sports/Recreation (53.5 vs. 51.3, *p* = 0.65) and Quality of life (37.6 vs. 36.7, *p* = 0.81). The average time from primary injury to surgery was 6 months for the ACL group and 31 months for the RLPD group.

**Conclusion:**

RLPD affected knee function as much as ACL deficiency, and was associated with more pain. Still the RLDP patients waited on average 5 times longer for surgery.

**Trial registration:**

The patients with RLPD consisted of patients who were examined for possible recruitment for a concurrent prospective randomized controlled trial comparing conservative treatment and isolated surgical medial patellofemoral ligament (MPFL) reconstruction (Clinical trials no: NCT02263807, October 2014).

## Introduction

Lateral patella dislocation is a common serious knee injury that occurs mostly among adolescents and young adults. Patients with lateral patella dislocation give reports of considerably reduced knee function, quality of life and pain compared to reference values for the same age group for many years after their primary event [1]. In addition, 17–42% are likely to experience further dislocations, which substantially increase the risk of developing subsequent instability problems, patellofemoral pain and decreased knee function [1, 2].

It is well-known that patients with patella dislocation form a heterogeneous group in terms of anatomic risk factors, dislocation frequency and injury mechanisms, and there is a frequent recurring discussion related to “who, when and how” in the treatment of these patients [3, 4]. Nevertheless, there seems to be an agreement in the literature that patients with recurrent lateral patella dislocations (RLPD) should be considered for surgery [5]. A significant factor endorsing surgical treatment is that these young patients lose confidence in the injured knee and experience symptoms and loss of function that compromises their level of activity or even their daily life functioning [1]. Patella stabilising surgery, and isolated medial patella femoral ligament (MPFL) reconstruction in particular, has started to show promising results with a high number of young patients returning to sport after surgery, and low incidence of recurrent instability [6].

This situation is comparable to another common knee injury within the same age group, the anterior cruciate ligament (ACL) rupture. The current recommended indication in Scandinavia for ACL reconstruction is persistent instability of the knee and a subjective feeling of give away after 6–12 weeks of functional rehabilitation and/or an activity level in demand of a good knee function [7–11]. Hence, surgical treatment of both RLPD patients and ACL deficient patients is based on persistent instability and the level of deteriorated knee function in these young patients. To some degree this is irrespective of the anatomical cause and therefore it would be interesting to compare the pre-operative knee-related symptoms and function between these two groups. Particularly since the time from injury to surgery reported for the RLPD patients seems to be significantly longer than what is known from the ACL register data [9, 12–17].

The purpose of this study was therefore to explore the time from injury to surgery and the pre-operative symptoms and knee function of young RLPD patients scheduled for stabilizing surgery and compare this group to age and sex-matched ACL-deficient patients booked for surgical reconstruction.

## Methods

### Study design

A cross-sectional study was performed using a pre-operative questionnaire to assess knee function and symptoms in young patients with RLPD and sex, age and BMI matched patients with ACL injury awaiting surgical reconstruction.

### Patients

The study recruited patients referred to the orthopaedic outpatient clinic at Akershus University Hospital for recurrent patella dislocations between May 2010 and December 2015. The patients with RLPD consisted of patients who were examined for possible recruitment for a concurrent prospective randomized controlled trial (RCT) comparing conservative treatment and isolated surgical medial patellofemoral ligament (MPFL) reconstruction (Clinical trials no: NCT02263807). The inclusion criteria listed in Table 1 were therefore based on the recommended indication for isolated MPFL reconstruction [5, 18]. Bilateral cases were excluded as the protocol for the prospective RCT included comparisons with the contralateral leg in the follow-ups. All patients eligible for isolated MPFL reconstruction were asked to fill in the baseline assessment forms on the day of surgery.

**Table 1:** Inclusion, exclusion and matching criteria for the patients with recurrent patella dislocation (RLPD group) and their ACL-deficient patients (ACL) in the study

Inclusion criteria for the RLPD group	
a. Patients with two or more patella dislocations	
b. Patients with a lateral dislocation of the patella and a positive apprehension test with clinical examination	
c. Patients aged between 12 and 30 years	
Exclusion criteria for the RLPD group	
a. Patients with medial dislocation	
b. Bilateral patella instability at inclusion	
c. Severe trochlea dysplasia grade D (Dejour)	
d. Tibal Tuberosity Trochlear Groove (TT-TG) distance > 20 mm on CT	
Inclusion criteria for ACL patients from the Norwegian Knee Ligament Registry (ACL)	
a. Patients with isolated ACL rupture verified both with clinical examination and MRI	
b. Patients aged between 12 and 30 years	
c. Knee symptoms indicating operative treatment	
d. Registered height and weight at time of operation	
Matching criteria	
a. Matched by sex, age and body mass index (BMI) in that order (more specific; best BMI match within +/− 1 year)	
b. Two controls per case - data truncated into one average score	

The ACL group were selected among patients with isolated primary ACL reconstruction (+/− meniscal procedures) registered in the Norwegian National Knee Ligament Registry (NKLR) from 2004 to 2012. The NKLR was established in June 2004 and all Norwegian hospitals performing ACL-reconstructions report to the registry with a compliance rate of approximately 80% [19]. The ACL patients were cross-matched with the RLPD patients based on sex, age and BMI. Each RLPD case was matched with two ACL patients.

### Outcome measures

The main outcome measure was time from primary injury to surgery and the Knee injury and Osteoarthritis Outcome Score (KOOS) filled in on the day of surgery by the the RLPD patients for this study and by the ACL patients as per protocol for the NKLR.

KOOS consists of 42 questions divided into 5 subscales; Pain, Symptoms, ADL (activities of daily living), Sport/Rec (function in sports and recreation) and QoL (knee related quality of life). Each question is scored from 0 (worst) to 4 (best) on a Likert scale, and a normalized score from 0 to 100 is calculated for each subscale which are independently used in all outcome comparisons, as recommended [20]. Calculation of the score of each subscale and missing data were treated according to the guidelines provided by Roos et al. [20].

The NKLR provides simple demographic data such as age, sex, height and weight, and corresponding data was also filled in pre-operatively by the RLPD patients. All RLPD patients also filled in their pre-injury Tegner activity score which is a one-item score from 0 to 10 where 0 represents disability due to the current knee condition and 10 represents competitive level in pivoting sports [21]. However, activity data was not registered in the NKLR for the ACL patients in the study period.

### Statistics

Statistical analyses were performed using SPSS 22 (SPSS Inc., Chicago, Illinois). A paired sample T-test was chosen using the aggregated mean KOOS score for the two sex, age and BMI matched ACL controls as pair for each one of the RLPD patients. This was performed in consensus with previously published studies using NKLR data [22, 23] in order to rule out known confounders for the KOOS score such as age and sex [24–26]. An independent T-test or the Chi Squared test was used to compare the demographic data according to type and distribution of the examined variable. The level of significance for all tests was defined as *p* < 0.05. Power analysis revealed that 18 pairs of patients were needed to detect a difference in KOOS QoL of 10 points (SD 15) with a power of 0.80 based on reference data from the developers of the KOOS [27].

## Results

**Fig. 1:**
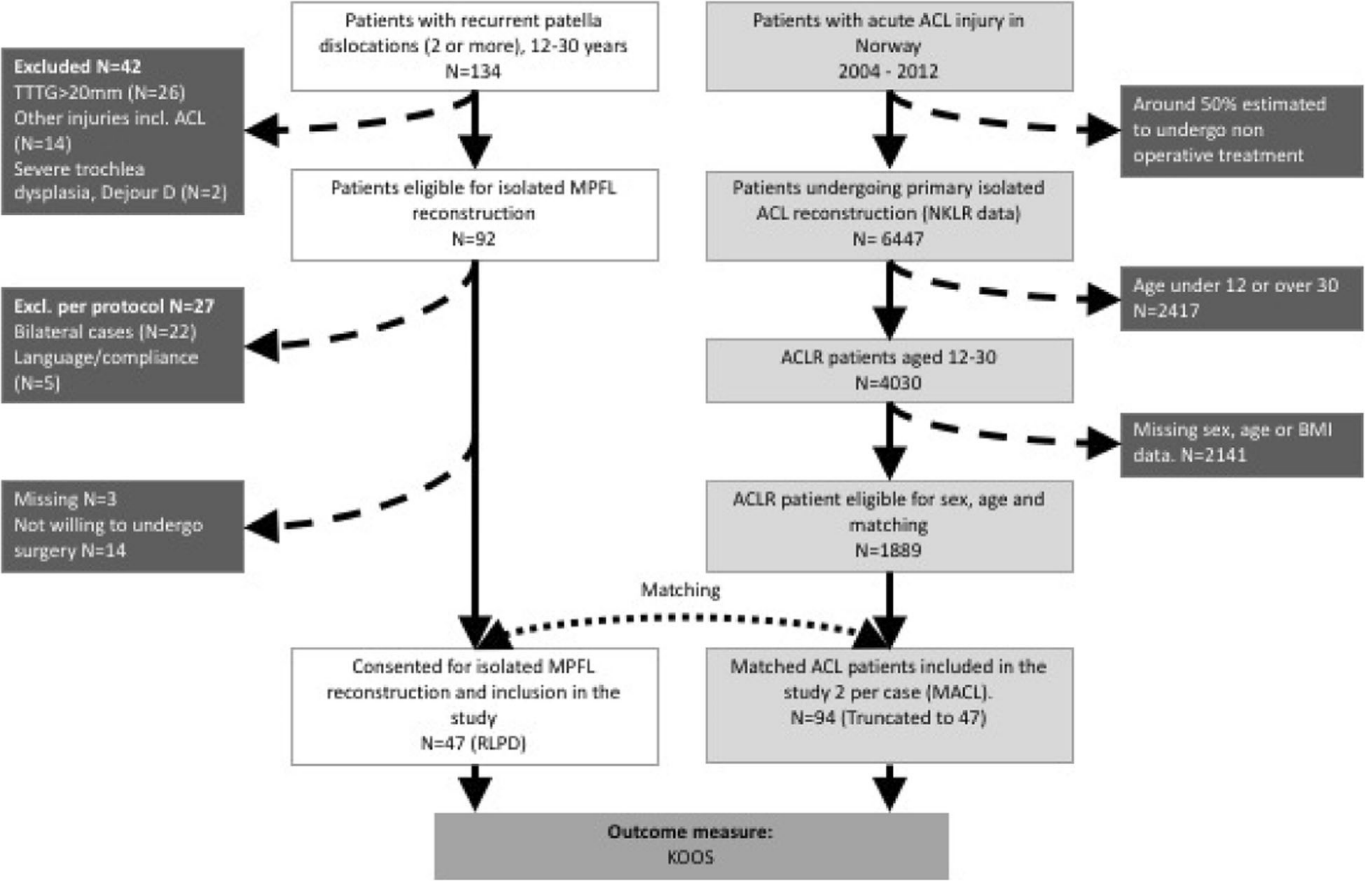
Flowchart of the inclusion of the different patient groups in the study

From May 2010 to December 2015, a total of 134 patients were referred to our outpatient clinic for suspected recurrent patella dislocations. As shown in Fig. 1, a total of 92 of these RLPD patients (68.7%) were found eligible for treatment with isolated MPFL reconstruction. Notably, only two cases (1.5%) were found to have a severe trochlear dysplasia as their main cause for RLPD and were referred for a trochleaplasty, and a total of 17 (12.7%) did not want any surgical treatment. After exclusion of bilateral cases and patients not able to complete forms due to language barriers (the forms were only available in English and Norwegian), 47 (51.1%) RLPD patients were finally included in the study. These 47 were matched with 94 ACL patients selected from the NKLR registry (Fig. 1). The demographics for both groups and all the ACL patients eligible for matching are presented in Table 2. For the RLPD group the mean Tegner activity score was 4.4 (SD 2.8) and 25 (53.2%) patients reported that their latest dislocating event happened during sporting activities, where football (*N* = 9, 19.1%) was most frequent.

**Table 2:** Demographics for the different patient groups in the study

	Recurrent lateral patella dislocation (*N* = 47)	Matched ACL-deficient patients (ACL) group (*N* = 94)	All eligible ACL patients from the ACL registry (NKLR) (*N* = 1889)
Age at time of operation/inclusion (mean, (SD))	19.2 (5.0)	19.5 (4.9)	21.4 (4.9)*
Time in months from injury to operation (median, (quartiles))	31 (16–69)*	6 (4–12)	6 (4–12)
BMI (mean, (SD))	23.2 (4.7)	23.1 (3.9)	24.0 (3.4)
Sex	M: 15 (31.9%)	M: 30 (31.9%)	M: 894 (47.3%)*
	F: 32 (68.1%)	F: 64 (68.1%)	F: 995 (52.7%)*

*Significantly different from the other groups, *p* < 0.05

**Fig. 2:**
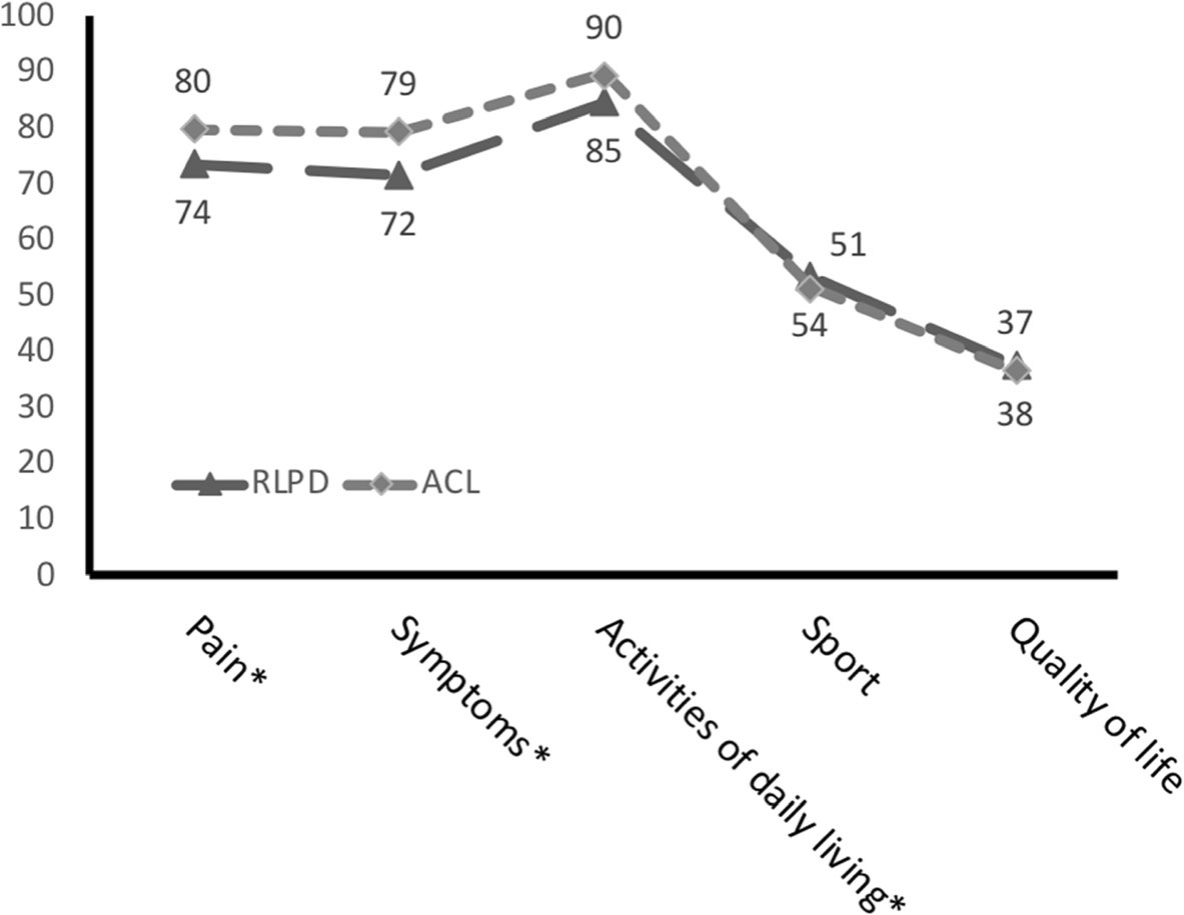
Preoperative KOOS score for Recurrent lateral patella dislocation group (RLPD, *N* = 47) and the matched ACL group (ACL, *N* = 94). *Significantly different from RLDP, *p* < 0.05

For the main outcome measure KOOS; the RLPD-group scored significantly lower than the ACL-group for the three subscales “Pain”, “Symptoms” and “ADL” (Fig. 2). The lowest KOOS values were found for the Sports/Recreation (Sports/Rec) and Quality of life (QoL), but here there was no significant difference between the two groups. However, median time in months from injury to operation was significantly longer for the RLPD group (31, quartiles 16–69) compared to the ACL-group (6 (quartiles 4–12).

## Discussion

This study found that patients with RLPD struggle with their knee injury for many years before surgery is attempted. Their KOOS subscores for the Sports and QoL function were particularly low, but these were at the same level as their age and BMI matched patients with ACL-deficient knees scheduled for surgery. In addition, the RLPD patients report worse scores for pain, knee related symptoms and ADL function compared to their ACL peers. It is therefore interesting that the RLPD patients had waited close to 2.5 years on average before they were considered for surgery, compared to the ACL patients where 50% were operated within 6 months after the injury.

One can argue that this discrepancy in time from injury to treatment might only be representative for the practise at the study hospital or region where these RPLD patients were recruited, but other studies have reported comparable numbers with time from first injury to surgery ranging from 1 to 7 years on average [12–17]. One obvious explanation is that there is a need for at least one re-dislocation to be considered as a patient with RLPD, and the time between the first and second event can vary from weeks to many years. However, the highest incidence of re-dislocation is reported to be within the first months, but the curve does not flatten out until after 2 years [28]. In contrast, for the ACL deficient patients the indication for surgery is usually based on persistent instability of the knee and subjective feeling of give away after 6–12 weeks of functional rehabilitation [7–11]. Furthermore, while the treatment and rehabilitation of the ACL deficient knees are well documented, the adequate approach for RLPD is still under debate [3–6]. However, recent reviews have shown that 84% of the patients return to sport after stabilising surgery for RLPD and the corresponding incidence of recurrent instability and reoperations were low [6, 29]. These results are comparable to what has been reported for ACL-reconstruction [30]. The best results are found for the RLPD patients where an isolated MPFL reconstruction is considered as sufficient, which was the case for the RLPD patients in this current study. Still, surgeons seems be more eager to propose a surgical treatment for ACL deficient patients than for those with RLPD.

One reason for this is that an ACL injury is known as a common “sports” injury and surgeons could be tempted to consider ACL injured patients as a group of patients with a higher activity level and a higher demand to their knee function, which in turn would trigger a recommendation for surgical reconstruction [31]. On the other hand, a larger number of the ACL patients may be high performance athletes which could be part of the explanation. They are high maintenance and seek surgical solution quickly, their teams have insurance and support and this brings them to the operating table much quicker than the RLPD patients. This study also observed that the pre-injury level of activity for the RLPD patients was indeed lower compared to the numbers presented in the literature for patients with an ACL injury [32]. However, even though it seems like the RLPD patients do not have as high sporting demands as compared to the ACL deficient patients, more that 50% of the young RLPD patients reported that their last dislocation occurred during sports activities. Further on, they also report that their reduced knee function reduces their quality of life to the same level as for the patients with an ACL injury. In addition, they report more pain and everyday knee-related symptoms.

When comparing the KOOS scores for the RLPD group to previous studies assessing patients after a primary lateral patella dislocation, this study observed lower scores for all KOOS components. Interestingly, in the study by Magnussen et al. [1], they found no effect of re-dislocations on the KOOS. None of the cases in their study underwent surgical treatment; hence their result could be affected by a subgroup of RLPD patients that were coping quite well. In the inclusion process of this current study, 17 out of the 64 RLPD patients who were eligible for inclusion, felt that they were coping well with their knee injury or at least were not interested in surgical treatment.

## Limitations

In contrast to most of the previous studies assessing symptoms and function in patients with RLPD [13, 33–35], the RLPD patients included in this study were the ones eligible for treatment with isolated MPFL reconstruction. This limits the generalisability of the KOOS findings, but according to previous studies assessing the risk of further patella dislocations, these patients were likely to represent the milder spectrum of the RLPD patients [4].

The KOOS itself assesses impairments without a specific patellar instability component. However, KOOS has successfully been used in several studies assessing this patient group [1, 36–38]. Further on, the main purpose of this study was not to assess the patellar instability, as all patients had known RLPD condition, but further to assess how well they were coping with this injury. In addition, the purpose was also to relate their impairments to a comparable group of ACL deficient patients, which would not be possible with a more patellofemoral-specific instrument. KOOS is a widely used and well-known instrument validated for assessing several different knee injuries and it has been reported to have the largest effect size in young active persons [27, 39].

## Perspectives

The findings of this current study emphasise the importance to identify the RLPD patients who are not coping as early as possible, as a delayed treatment of these patients seems to result in a sustained inability to trust the knee as well as anxiety related to the fear of re-dislocation, which might prevent further participation in sports and recreational activities [2, 40]. Accordingly, a more thorough primary review of the patients with lateral patella dislocation is recommended in order to early identify those who are likely to re-dislocate, and further on to consider a routine follow-up (i.e. after 6–12 weeks) similar to the recommended follow-up routine which takes place after an isolated ACL rupture in Scandinavia [7, 8, 31]. Preferably, after a standardised rehabilitation programme focusing on stronger quadriceps (vastus medialis obliquus), stability training of the knee and hip, and protective bracing and/or taping to prevent early re-dislocations [3]. The goal must be to prevent that the patients with a primary lateral patella dislocation turn into RLPD patients and to identify the ones who do as early as possible.

## Conclusion

Young patients with RLPD reported that their knee condition significantly reduced their sports function and quality of life to the same level as for their ACL-deficient peers. In addition, they experienced more pain, knee-related symptoms and restrictions in their activities of daily living. Still the RLDP patients wait on average 25 months longer for surgery.

## Data Availability

The datasets used and analysed for this current study are available from the corresponding author on reasonable request.

## Abbreviations

ACL: Anterior cruciate ligament
ADL: Activities of daily living
BMI: body mass index
KOOS: Knee injury and Osteoarthritis Outcome Score
MPFL: medial patellofemoral ligament
NKLR: Norwegian National Knee Ligament Registry
RCT: Randomized controlled trial
RLPD: recurrent lateral patella dislocation
TT-TG: Tibal Tuberosity Trochlear Groove
